# Military medicine in China: old topic, new concept

**DOI:** 10.1186/2054-9369-1-2

**Published:** 2014-04-15

**Authors:** Xiao-Bing Fu

**Affiliations:** Key Laboratory of Wound Repair and Regeneration of PLA, College of Life Sciences, General Hospital of PLA, Medical College of PLA, 28 Fu Xing Road, Beijing, 100853 P.R. China

**Keywords:** Military medicine, Surgery, Military epidemiology, Sanitary science, China

## Abstract

Military medicine is important in both war and peace. In China, military medicine plays a key role in supporting and maintaining health, in preventing injuries and diseases in military staff and in enhancing the military armed forces during war. Additionally, military medicine participates in actions such as emergency public health crises, natural disasters, emerging conflicts and anti-terrorist campaigns during peacetime. In this paper, we summary the current condition and achievements in military medicine in China and provide our perspective for its future.

## Background

Military medicine is an important field in biological and medical sciences. Military medicine plays a key role in supporting and maintaining health, in preventing injuries and diseases in military staff and in enhancing the military armed forces during war [1]. In the new millennium, military activities also involve other actions such as emergency public health crises, natural disasters, emerging conflicts and anti-terrorist campaigns during peace. Thus, military medicine has faced new challenges and requirements with the military transformation. These requirements include medical services in natural disasters, accidents, terrorist attacks, and in epidemiological diseases. Thus, the topic is old but has a new concept in a rapidly developing society. Accompanying the new military activities, such as the Chinese naval forces on escort duties in the Gulf of Aden, international peacekeeping activities and humanitarian relief, the military medical mission will have more responsibility in important areas.

### Military surgery and internal medicine

The armed forces are affected by many elements, such as military equipment, technology, organization and human resources; however, the most important element is “people”. Maintaining physical and mental health is the fundamental element of reinforcing the armed forces, rapidly recovering from injury, and of regenerating the fighting capacity. The aim of military medical research is to serve the people and to manage people-related health problems. Traditionally, injury from weapons is called “trauma” but can be associated with many serious complications and can cause systematic reactions and internal organ damage. These damages are the central part of war injuries but has similarities with diseases in usual healthcare; sometimes these damages are called disease trauma, injury and illness, as well as injury complicated with disease. Modern war can not only create somatic trauma and associated diseases but also involve psychological trauma. Therefore, military medicine refers to traditional military field surgery or internal medicine as well as the overall health of military staff, particularly how to manage mental stress. Currently, military surgery and internal medicine research emphasize the reform of first aid skills and equipment, regenerative medicine, etc.

In March 1999, the People’s Liberation Army (PLA) Military Medical Institute (MMI) established the Military Stress Research Department, which began many research projects in this area and has had many accomplishments. Under the current “only child” policy in China, this area has become an important issue in military medicine, covering issues such as how to consult for mental health and how to establish psychological-stress standard measurements and management guidelines [2].

Military medicine can be called translational medicine or a branch of translational medicine [3]. Using the methods of biology, pathophysiology, targeting pharmacology, pharmacological genetic phenotyping, and genetic phenotyping, military medicine can introduce new theories, concepts, and technology into basic research areas, such as epidemiology, pathogenesis and pathological response, mechanisms of complications after injury, regeneration after severe organ damage, mental stress, and psychological responses to somatic and mental trauma. The ultimate goal is to provide services in detection, prevention, diagnosis, and treatment.

### Sanitary science and prevention medicine in extreme environments and in special military operation fields

Military activities, such as guarding, training, fighting, and surviving, are usually performed in special environments. The environment plays an important role in the health of military staff. The environment can mean the natural environment (such as remote mountain areas, tropical areas, highlands, oceans, deserts, epidemic or space areas); human-made environments (such as closed, high-pressure, weightless environments); information environments (space fields with information or information-related launching, transmitting, utilizing media carriers); and mental environments (mental stress and mental health-related). Special operations should also include tasks in space, aviation, navigation, and underwater diving. The damage to humans in these areas is usually multi-factorial and more complicated and serious than single-factor damage.

For historical reasons, high-altitude–related military medical research is the strong point in Chinese military medicine, with many accomplished projects, such as the study of the effect and recovery of systemic or organic hypoxia and the mechanism of diseases in highlands. More than 10 diagnostic criteria and management guidelines for high-altitude reactions and hypoxia coma have been developed, as well as a high-altitude standard sanitary system [4]. In addition, military aviation medicine was established in 1949. Many types of aviation medical equipment have been developed by the military service in China in relation to much basic and applied research into mental health, aviation physiological training, pilot selection and unhealthy effects from hazardous aviation environments [5]. Because the space program in China has been established for more than 40 years, aviation medical-engineering technology has rapidly developed and now has more than 13 branches, including aviation environmental medicine, cell biology, and psychiatry [6]. A few branches, such as cardiology, have remained in a leading position in the world; however, some branches, such as aviation environment and psychiatry, remain behind the international level.

For military operations under a full-dimension environment, military medicine in China is now expanding its role in the field, reinforcing research into the military environment and into human–machine cooperation. Military medical research will potentiate the capability of human effectiveness and endurance under special situations and operations, including physical fitness, skillfulness, intelligence, human–machine cooperation, emotional perception and overall military operational capability, as well as survival in extreme environments and the enhancement of initiatives.

### Preventing and managing injuries due to high-technology and new-concept weapons

Because of the rapid development of high-technology, new-concept, and special weapons, the prevention and management of injuries from these weapons are essential for military medicine. New-concept weapons have new injury mechanisms beyond traditional injury mechanisms. Revolutionized weapons can also increase the range of human post-traumatic response, creating more difficult and complicated situations for treatment. In the research areas of damage from nuclear, chemical, and biological weapons and of secondary injury from these weapons, China has established standard “triple-prevention” rescue guidelines, including equipment and basic rescue techniques. In the area of detecting, preventing and managing microbiological agents, military medical research in China has reached the international level but should continue developing specialized military supply systems with minimal side effects. However, because of the restrictions in international law and arms control, the possibility of using nuclear, chemical, and biological weapons is small. Sub-nuclear weapons (uranium bombs, powerful fuel air explosives, electromagnetic pulse weapons, deep penetrator weapons, and metal hydrogen weapons) or fourth-generation sub-nuclear, sub-chemical, and sub-biological weapons (such as incapacitating agents and detonation inhibitors) will bring more healthcare problems.

During the Shanghai APEC meeting, the Beijing Olympics, the World Expo 2010 in Shanghai, China and the Guangzhou Asian Games, the Chinese military “triple-prevention” medical rescue team played a key role in safeguarding and had great contributions to these conventions [7].

Military medicine in China will continue to research the prevention and management of injuries caused by high-technology, new-concept and newly developed nuclear and chemical weapons. Injuries from these weapons include pathophysiological, serious, multi-organ, multi-location, combined, direct and indirect injuries; diseases during wartime; secondary psychiatric trauma and somatic trauma complicated with psychiatric trauma; and long-term effects. Simultaneously, military medicine must accelerate research into medical rescue, the production of presentational drugs, storage, equipment, and fast-detecting equipment and build up the basic laboratory research platform.

### Military epidemiology

Epidemiologic prevention is an important field in military medicine. Military staff must be treated as a special group, with a close association with not only diseases in the entire country and society but also characteristics of confined living conditions, a rapidly changing population and extreme living conditions, such as remote mountains and oceans. The pathologies of such staff greatly differ from that in the general population, particularly in epidemiologic incidence and spreading. In terms of biological and microbiological attacks, epidemiologic diseases will spread quickly. In addition to working hard in regular healthcare, researchers must reinforce research into epidemiological prevention to ensure no major outbreaks of disease occur during or after war.

Coartem, which is a drug for malaria that was invented by the MMI, is one of three patented drugs listed by the World Health Organization (WHO) in 28 years. This drug has saved the lives of more than 1 million malaria patients. During the 2003 SARS epidemic, researchers in the MMI first isolated and genetically analyzed the SARS virus. In 2008, severe freezing occurred because of cold weather in southern China; many people were saved by a lotion invented by the MMI and were airdropped a manual to prevent freezing. In March 2009, the epidemic of bird flu H1N1 occurred worldwide, and the military health organization rapidly adopted emergency actions, such as inventing a fast pathogen-detection system, epidemic surveillance, provision of preventive drugs and equipment. Diagnostic kits for H1N1 were invented, as were other management systems, such as diagnostic, prevention and treatment guidelines. The military disease-control organization joined the national public health system in 2005, which increased the opportunities for military preventive medicine [8].

Recently, with the help of the development of the military health care system, cooperation, wide application of new material, new technologies, and of new standards, military medicine has developed research into new equipment and reforming tasks. Much large health equipment, such as sanitary trains, airplanes and war-zone hospitals, appeared in the healthcare and supply competition in 2009 to connect highways, railroads, and air medical rescue transportation and to establish a full-dimension new supply system, further improving the supply and support system. Six healthcare support systems were created: emergency rescue, full-dimension transportation, the prevention of nuclear-biological weapons, rapid response, and healthcare information technology and environmental protection under special situations. The largest navy hospital warship, “Peace Ark,” which was equipped with an ambulance boat, escort helicopter, and shipboard medical modular, established a modern medical treatment and rescue platform on the ocean for systemic search and rescue, management, and transportation. By connecting information technology between the land, sea, and air, a full-dimension healthcare system has been established with high-level technology and information and improvements from single to multiple elements, from loose-constructed to integrated construction, and from equipped mobile to mobile-equipped.

Currently, the efficiency and quality of the epidemiological surveillance system still must be improved. Simultaneously, scientists should accumulate experience in preventing epidemiological diseases adapted to preventive medicine’s theory and technology system to help the public and to form the basis for future military medical activities. From the requirements for healthcare equipment from modern war, scientists must upgrade the capacity of the supply system. In the future, the multi-functional and modular system must focus on improving the emergency-response mobile capacity, with the function of improved intellectualization, information, and mimicking war zones.

### Information and networked healthcare system

With fast-developing internet technology, newly developed practical, high-efficiency, and full-dimension information technology will be the future direction of the military medical information technology system. Digitalized medical information technology has been popularized at each level of the military system. From the overseas developed technology and experiences, combined with the actual military situation in China, military medicine has begun to build its own medical information system, with military characteristics, serving its modern national defense system and with the ability to win any regional war. In 1988, the General Hospital of PLA held its first clinical-case telephone conference with a hospital in Germany. In 1995, the Health Department of the General Logistics Department proposed the project of “Army 1, 2, 3”, with 2 denoting the military medical information technology network and tele-medical consulting system. Currently, the number of satellite telecommunication systems has reached more than 90, including telecommunications in Tibei, Xinjiang, and Xisha, which are remote areas. Each large military district has built a telecommunications center in the central and regional district hospitals and in basic medical clinics, as well as a telecommunications working station on ocean research vessels, which have completed many tasks including medical service in the first manned flight of Shenzhou in 2000.

Future war will have multiple levels and mutual exchanges and will bring more difficulties to the military supply system. How to immediately transport injured military staff to the hospital behind the frontline, which has life-saving capabilities and many military medical experts, is an important issue. Because of the variety of military tasks, many army forces have to live in remote areas, such as mountains, oceans, and islands with poor live conditions. With the development of digitalized information technology, many medical resources can be shared with those remote areas, thus diminishing the differences between the remote areas and large cities and helping doctors and medical services with high-level medical equipment and experts.

### Military medicine in non-military rescue activities

The China International Rescue Team was established in April 2001 by the Beijing Military Engineering Department, which consisted of earthquake experts, technicians and medical rescue experts. The team can also play an important role in non-military activities, such as protection, non-military tasks, and the maintenance of social security.

During the Wenchuan earthquake medical rescue, the military force sent 103 medical teams and 3,167 medical staff, including 47 teams of psychiatrists. In 2009, the China Medical Rescue Association was established. In 2009, the Military Police Medical College founded the first medical rescue medicine department (MD degree). Many universities followed this lead and established their own medical rescue class. In 2009, textbooks on disaster rescue medicine were published. Many rescue guidelines and standards, including a national earthquake rescue training center, were set up. On August 8, 2010, a severe mud-rock flow occurred in Zhouqu in Gansu Province. On April 4, 2010, during the strong earthquake in Yushu, Qinghai Province, the army rescue team was sent, and the rescue response was rapid and organized, acting as the main force and lifesaving “Green Ark”.

Via mutual or multiple channels, China has provided humanitarian assistance to many countries such as in Asia, Africa, and the Caribbean, in an emergency crisis. In recent years, the Chinese Army has sent 4 second-degree peacekeeping and medical teams to the Congo, Libya, Lebanon, and Sudan. China and Gabon hosted a joint medical rescue action, called “Peace Angel 2009”, which was the first example of a joint medical training exercise. The Chinese army also sent a medical assistant team to joint international humanitarian exercises in Indonesia (2004 and 2006); Haiti (2010); Pakistan (2010); Japan (2011) and the Philippines (2013). In 2012, China and the United States held a joint humanitarian rescue and disaster diminishing exercise, “Mimic Exercise on the Table”. In June 2013, the Chinese military medical assistant team joined the humanitarian assistance and disaster relief/military medicine combined exercise of the ASEAN defense ministers meeting plus expert working groups and received praise due to its excellent performance.

Because China is playing roles that are more important in international activities, China has routinely sent international peacekeeping forces or military medical assistant teams to disaster areas. China will keep joining these international activities, which can provide precious practical and training opportunities for military medical assistant forces [9]. Military medicine is responsible for homeland security and national safety, such as nuclear crisis rescue, emergency public accidents, biologic weapons control, international peacekeeping and medical assistance, and anti-terrorist medical assistance. Military medicine must be brought to general guidelines and projects of homeland security, national safety, economy and strategy. To gain support for the protection of law and funding or for future development, military medicine should be legalized [10].

### Medical assistance organization

In 1997, the US army proposed the concept of force health protection and full-dimension protection, emphasizing improving health, protection and treatment of diseases and war-zone medical rescue or full protection, regeneration, and improvement of armed forces capability [11]. Full-dimension medical assistance has both general and special aspects. The general aspect involves full-dimension threats or injury (from natural, information, psychiatric, or social environments) and full-dimension health maintenance (before, during, and after serving the army, preventing emergency injury and its later effects, maintaining armed forces, the work force and living ability). Medical service will be under the direction of military, political, and logistics leaders cooperating with local government and with local medical or related resources. The abovementioned issues involve theories and practical applications, which require further investigation to improve and to establish a new medical assistance service theory and organization system to keep pace with the new military revolution.

Current research in military medical service is thorough, and its design is complete. However, non-war military medical service concerning anti-terror, peacekeeping, dealing with crisis, maintaining public safety, and disaster rescue is not systemic or is absent. Our plan of dealing with crises is too principled, conceptive, and general and lacks targeted, practical, applicable, or doable guidelines. Our military medical research still lacks a good design and is underdeveloped without strong associated research.

### Combining high-technology and traditional medicine in military medicine

New developments, such as genetic, cell, enzyme, and protein engineering, as well as genetic-chip, space-information, and nanometer technology, will bring rapid developments for medical research. Developments in neuron injury, space medicine, biology, radiology and radiation injury will provide important tools for military medicine research. These new development can help to develop new foundations for military medicine, to establish new research models, and to accomplish many special tasks under the new situation. These advanced and new technologies will change military medicine toward diagnosis and treatment as well as toward detection and prevention. The establishment of mimicked human technology and military medical biological engineering provide practical methods for researching medical rescue, medical assistance, the maintenance of armed forces health, and the enhancement of the effectiveness of the army supply system and for increasing the military capacity of armed forces [12].

Biotechnology can create new improvements in life information resources, micro-space and bio-control technology, involving the most important military information, such as ethnic genetic coding, functional control key, and creating new battling territory, with the ability of direct application to military defense attack systems and having the characteristics of “micro, defined, not lethal, and reversible”. Biotechnology can target changes in micro-structures and further define the control of special physiological functions to limit the injury of biological targets without lethal effects. Medical research into these issues can help develop military medicine.

Under the current requirements for military drug research and national medical strategy, scientists and doctors should disseminate the knowledge of traditional medicine among every level of the military forces. Scientists and doctors should find and develop military drugs from traditional medicine and must improve the survival ability of soldiers and officers, maintaining their mental health and combat force and improving military medical storage strategies. Using modern pharmacological research methods, such as molecular biology, molecular pharmacology, bio-neurology, bio-physics, cell biology, natural medical chemistry and computer science, scientists should increase the intensity of research into traditional medicine, focusing on selecting traditional drugs that fit the requirements of modern war.

## Summary

Establishing a policy of military medicine is the guarantee of the new military medicine environment and revolutionized system. Establishing and completing military medical policy will move military medicine in the correct direction of scientific development. Scientists should base their policy on the requirements of future war, through appropriate cooperation with civilian research resources, emphasizing the selection and education of a special military medical branch, with deepening and widening directions. In 2010, a proposal concerning the further deepening of military–civilian cooperation was published by the Ministry of Health; the General Logistics Department of PLA; education, health, and national traditional medicine departments; the National Food and Drug Control Administration; the China Science Institute; and by the China Academy of Engineering. Scientists must rely on national resources in developed and advantaged areas to widen the range of military medical research with full-dimension development. To form a solid base for future medical research, scientists also must attract military or civilian researchers and experts.

## Abbreviations

PLA: People's liberation army
MMI: Military medical institute
WHO: World health organization.
